# Pyridine-2,5-diamine

**DOI:** 10.1107/S1600536812046260

**Published:** 2012-11-17

**Authors:** Sergiu Draguta, Victor N. Khrustalev, Marina S. Fonari, Mikhail Yu. Antipin, Tatiana V. Timofeeva

**Affiliations:** a D. Ghitu Institute of Electronic Engineering and Nanotechnologies, 3/3 Academy str., MD-2028 Chisinau, Moldova; b X-Ray Structural Centre, A.N. Nesmeyanov Institute of Organoelement Compounds, Russian Academy of Sciences, 28 Vavilov St, B-334, Moscow 119991, Russian Federation; c Institute of Applied Physics Academy of Sciences of Moldova, 5 Academy str., MD-2028 Chisinau, Moldova; d Department of Chemistry & Biology, New Mexico Highlands University, 803 University Avenue, Las Vegas, NM 87701, USA

## Abstract

In the title mol­ecule, C_5_H_7_N_3_, intra­cyclic angles cover the range 117.15 (10)–124.03 (11)°. The N atoms of the amino groups have trigonal–pyramidal configurations deviating slightly from the pyridine plane by 0.106 (2) and −0.042 (2) Å. In the crystal, the pyridine N atom serves as an acceptor of an N—H⋯N hydrogen bond which links two mol­ecules into a centrosymmetric dimer. Inter­molecular N—H⋯N hydrogen bonds between the amino groups further consolidate the crystal packing, forming a three-dimensional network.

## Related literature

For general background, see: Domenicano *et al.* (1975 ▶); Domenicano & Vaciago (1979 ▶); Mootz & Wussow (1981 ▶); Crawford *et al.* (2009 ▶). For the crystal structures of isomeric diamino­pyridines, see: Schwalbe *et al.* (1987 ▶); Rubin-Preminger & Englert (2007 ▶); Al-Dajani *et al.* (2009 ▶); Betz *et al.* (2011 ▶).
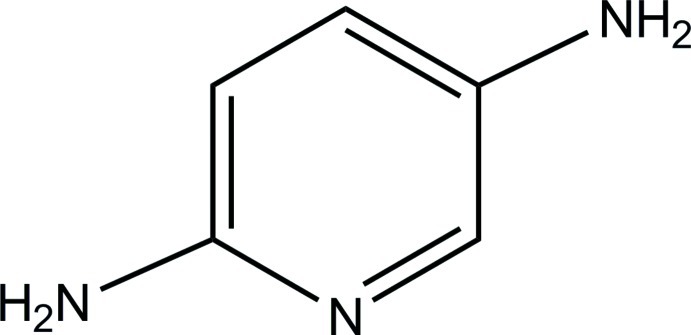


## Experimental

### 

#### Crystal data

C_5_H_7_N_3_
*M* *_r_* = 109.14
Orthorhombic, 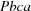

*a* = 11.4447 (11) Å
*b* = 7.1447 (7) Å
*c* = 12.8030 (12) Å
*V* = 1046.89 (17) Å^3^
*Z* = 8
Mo *K*α radiation
μ = 0.09 mm^−1^
*T* = 296 K
0.30 × 0.25 × 0.20 mm

#### Data collection

Bruker APEXII CCD diffractometer
Absorption correction: multi-scan (*SADABS*; Sheldrick, 2003 ▶) *T*_min_ = 0.973, *T*_max_ = 0.982
13022 measured reflections
1595 independent reflections
1240 reflections with *I* > 2σ(*I*)
*R*_int_ = 0.049

#### Refinement

*R*[*F* ^2^ > 2σ(*F* ^2^)] = 0.042
*wR*(*F* ^2^) = 0.109
*S* = 1.01
1595 reflections
85 parameters
H atoms treated by a mixture of independent and constrained refinement
Δρ_max_ = 0.36 e Å^−3^
Δρ_min_ = −0.24 e Å^−3^

### 

Data collection: *APEX2* (Bruker, 2005 ▶); cell refinement: *SAINT* (Bruker, 2001 ▶); data reduction: *SAINT*; program(s) used to solve structure: *SHELXTL* (Sheldrick, 2008 ▶); program(s) used to refine structure: *SHELXTL*; molecular graphics: *SHELXTL*; software used to prepare material for publication: *SHELXTL*.

## Supplementary Material

Crystal structure: contains datablock(s) global, I. DOI: 10.1107/S1600536812046260/cv5359sup1.cif

Structure factors: contains datablock(s) I. DOI: 10.1107/S1600536812046260/cv5359Isup2.hkl

Supplementary material file. DOI: 10.1107/S1600536812046260/cv5359Isup3.cml

Additional supplementary materials:  crystallographic information; 3D view; checkCIF report

## Figures and Tables

**Table 1:** Hydrogen-bond geometry (Å, °)

*D*—H⋯*A*	*D*—H	H⋯*A*	*D*⋯*A*	*D*—H⋯*A*
N2—H2*A*⋯N1^i^	0.874 (17)	2.183 (17)	3.0541 (15)	175.1 (10)
N2—H2*B*⋯N3^ii^	0.879 (17)	2.309 (17)	3.1457 (16)	159.3 (10)
N3—H3*A*⋯N2^iii^	0.894 (16)	2.397 (17)	3.2150 (16)	152.2 (10)
N3—H3*B*⋯N2^iv^	0.898 (17)	2.593 (17)	3.4803 (16)	170.0 (10)

Symmetry codes: (i) 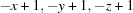; (ii) 
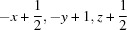
; (iii) 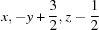; (iv) 
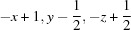
.

**Fig. 1.**
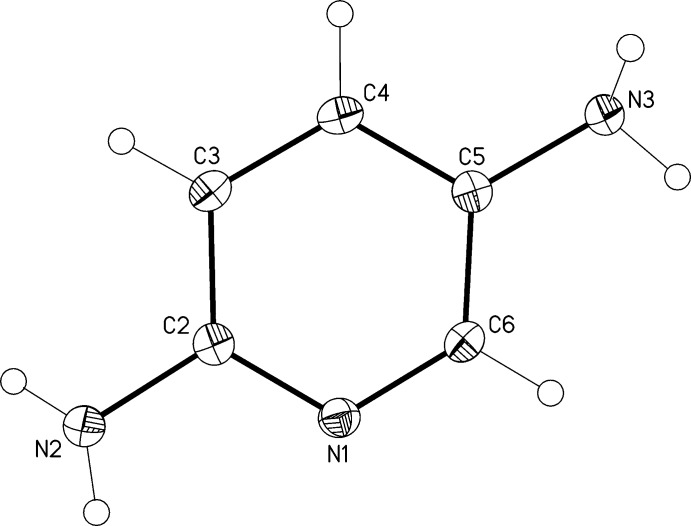
Molecular structure of I. Displacement ellipsoids are shown at the 50% probability level. H atoms are presented as small spheres of arbitrary radius.

**Fig. 2.**
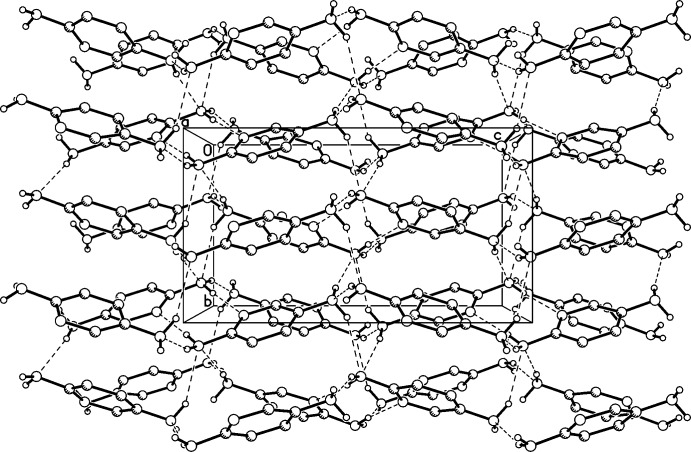
A portion of the crystal packing showing intermolecular N—H···N hydrogen bonds as dashed lines.

## supplementary crystallographic information

### Comment

Polydentate ligands have found widespread use in coordination chemistry due to the increased thermodynamic stability of resultant coordination compounds as compared to that of such compounds with monodentate ligands. In this aspect, the title compound can be considered as a versatile polydentate ligand in metal-organic synthesis. Furthermore, owing to the presence of three donor atoms, the title compound might play a role of the building block in the formation of metal-organic frameworks as well as for cocrystals.

In this work, we determined the crystal structure of the title compound (I), C_5_H_7_N_3_ (Figure 1), to enable comparative studies of its geometrical parameters in metal-organic complexes. Yet, the crystal structure of I can be helpful in the future investigations.

The geometry of aromatic molecules is known to be sensitive to the electronic effects of substituents. Based on the crystallographic analysis of monosubstituted arenes, it was concluded (Domenicano *et al.*, 1975; Domenicano & Vaciago, 1979) that the endocyclic angle at the *ipso*-C atom is > 120° for a σ-electron-withdrawing substituent and < 120° for a σ-electron-donating substituent. Moreover, in the nitrogen-containing heterocyclic aromatic molecules, the endocyclic angle at the nitrogen atom is < 120°, and those at the carbon atoms in *ortho*-positions to the heteroatom are > 120° (Mootz & Wussow, 1981; Crawford *et al.*, 2009). Similarly to the related diaminopyridines (Schwalbe *et al.*, 1987; Rubin-Preminger & Englert, 2007; Al-Dajani *et al.*, 2009; Betz *et al.*, 2011), these effects are also manifested in the investigated compound. So, the smallest endocyclic angle in I (117.13 (10)°) is observed at the C5 carbon atom bearing the σ-electron-donating amino group, whereas the largest endocyclic angle in I (124.03 (11)°) is observed at the C6 carbon atom in *ortho*-position to the N1 heteroatom. The value of the endocyclic angle at the second (C2) carbon atom in *ortho*-position to the N1 heteroatom (121.83 (11)°) is significantly smaller than that at the C6 carbon atom because the C2 carbon atom bears the σ-electron-donating amino group, *i.e.*, the C2 carbon atom is subjected to the influence of the two opposed electronic effects. The value of the endocyclic angle at the N1 heteroatom (118.15 (10)°) is also in good agreement with the above mentioned electronic effects.

The N2 and N3 nitrogen atoms of the amino groups have the trigonal-pyramidal configurations. It is worthy to note that these atoms are slightly out of the plane defined the aromatic system (r.m.s. deviation is 0.003 Å) by 0.106 (2) and -0.042 (2) Å, respectively. Apparently, this fact is explained by the developed hydrogen bonding system with the participation of the both amino groups.

In the crystal of I, the amino groups act both as proton donors and proton acceptors upon formation of the intermolecular N—H···N hydrogen bonds (Table 1). The N1 heteroatom serves as the acceptor for a hydrogen atom of one of the two amino groups (Table 1). In total, the molecules of I are linked by the intermolecular N—H···N hydrogen bonds into a three-dimensional network (Figure 2). There are no π-π interactions between the aromatic rings.

### Experimental

The compound I was obtained commercially (Aldrich) as a fine-crystalline powder and purified additionally by filtration. Crystals suitable for the X-ray diffraction study were grown by slow evaporation from chloroform solution.

### Refinement

The hydrogen atoms of the amino groups were localized in the difference-Fourier map and refined isotropically with fixed isotropic displacement parameters [*U*_iso_(H) = 1.2*U*_eq_(N)]. C-bound H atoms were placed in calculated positions [C—H = 0.93 Å], and refined as riding, with *U*_iso_(H) = 1.2*U*_eq_(C).

### Crystal data

**Table:**

C_5_H_7_N_3_	*F*(000) = 464
*M**_r_* = 109.14	*D*_x_ = 1.385 Mg m^−^^3^
Orthorhombic, *P**b**c**a*	Mo *K*α radiation, λ = 0.71073 Å
Hall symbol: -P 2ac 2ab	Cell parameters from 2334 reflections
*a* = 11.4447 (11) Å	θ = 3.2–28.8°
*b* = 7.1447 (7) Å	µ = 0.09 mm^−^^1^
*c* = 12.8030 (12) Å	*T* = 296 K
*V* = 1046.89 (17) Å^3^	Prism, red
*Z* = 8	0.30 × 0.25 × 0.20 mm

### Data collection

**Table:**

Bruker APEXII CCD diffractometer	1595 independent reflections
Radiation source: fine-focus sealed tube	1240 reflections with *I* > 2σ(*I*)
Graphite monochromator	*R*_int_ = 0.049
φ and ω scans	θ_max_ = 30.5°, θ_min_ = 3.2°
Absorption correction: multi-scan (*SADABS*; Sheldrick, 2003)	*h* = −16→16
*T*_min_ = 0.973, *T*_max_ = 0.982	*k* = −10→10
13022 measured reflections	*l* = −18→18

### Refinement

**Table:**

Refinement on *F*^2^	Primary atom site location: structure-invariant direct methods
Least-squares matrix: full	Secondary atom site location: difference Fourier map
*R*[*F*^2^ > 2σ(*F*^2^)] = 0.042	Hydrogen site location: difference Fourier map
*wR*(*F*^2^) = 0.109	H atoms treated by a mixture of independent and constrained refinement
*S* = 1.01	*w* = 1/[σ^2^(*F*_o_^2^) + (0.0475*P*)^2^ + 0.63*P*] where *P* = (*F*_o_^2^ + 2*F*_c_^2^)/3
1595 reflections	(Δ/σ)_max_ < 0.001
85 parameters	Δρ_max_ = 0.36 e Å^−^^3^
0 restraints	Δρ_min_ = −0.24 e Å^−^^3^

### Special details

**Table:**

Geometry. All e.s.d.'s (except the e.s.d. in the dihedral angle between two l.s. planes) are estimated using the full covariance matrix. The cell e.s.d.'s are taken into account individually in the estimation of e.s.d.'s in distances, angles and torsion angles; correlations between e.s.d.'s in cell parameters are only used when they are defined by crystal symmetry. An approximate (isotropic) treatment of cell e.s.d.'s is used for estimating e.s.d.'s involving l.s. planes.
Refinement. Refinement of *F*^2^ against ALL reflections. The weighted *R*-factor *wR* and goodness of fit *S* are based on *F*^2^, conventional *R*-factors *R* are based on *F*, with *F* set to zero for negative *F*^2^. The threshold expression of *F*^2^ > σ(*F*^2^) is used only for calculating *R*-factors(gt) *etc*. and is not relevant to the choice of reflections for refinement. *R*-factors based on *F*^2^ are statistically about twice as large as those based on *F*, and *R*- factors based on ALL data will be even larger.

### Fractional atomic coordinates and isotropic or equivalent isotropic displacement parameters (Å^2^)

**Table:**

	*x*	*y*	*z*	*U*_iso_*/*U*_eq_	
N1	0.46050 (8)	0.50612 (15)	0.35845 (8)	0.0150 (2)	
N2	0.36236 (10)	0.66538 (15)	0.49098 (8)	0.0167 (2)	
H2A	0.4111 (14)	0.610 (2)	0.5333 (13)	0.020*	
H2B	0.2925 (15)	0.674 (2)	0.5190 (12)	0.020*	
N3	0.38898 (10)	0.41571 (15)	0.07954 (8)	0.0164 (2)	
H3A	0.3841 (13)	0.510 (2)	0.0337 (12)	0.020*	
H3B	0.4559 (15)	0.352 (2)	0.0701 (12)	0.020*	
C2	0.36402 (10)	0.59703 (16)	0.38919 (9)	0.0139 (2)	
C3	0.27102 (10)	0.63298 (17)	0.32018 (9)	0.0148 (2)	
H3	0.2045	0.6952	0.3433	0.018*	
C4	0.28001 (10)	0.57463 (16)	0.21780 (9)	0.0150 (2)	
H4	0.2192	0.5971	0.1712	0.018*	
C5	0.38092 (10)	0.48140 (16)	0.18414 (9)	0.0139 (2)	
C6	0.46769 (10)	0.45101 (17)	0.25771 (9)	0.0148 (2)	
H6	0.5350	0.3889	0.2364	0.018*	

### Atomic displacement parameters (Å^2^)

**Table:**

	*U*^11^	*U*^22^	*U*^33^	*U*^12^	*U*^13^	*U*^23^
N1	0.0139 (5)	0.0159 (5)	0.0154 (5)	−0.0001 (4)	−0.0006 (4)	0.0002 (4)
N2	0.0162 (5)	0.0189 (5)	0.0150 (5)	0.0022 (4)	−0.0009 (4)	−0.0008 (4)
N3	0.0163 (5)	0.0190 (5)	0.0137 (5)	0.0027 (4)	0.0005 (4)	0.0003 (4)
C2	0.0148 (5)	0.0120 (5)	0.0148 (5)	−0.0018 (4)	0.0007 (4)	0.0006 (4)
C3	0.0130 (5)	0.0138 (5)	0.0176 (5)	0.0010 (4)	0.0003 (4)	0.0012 (4)
C4	0.0134 (5)	0.0138 (5)	0.0177 (5)	0.0001 (4)	−0.0024 (4)	0.0018 (4)
C5	0.0148 (5)	0.0124 (5)	0.0143 (5)	−0.0013 (4)	0.0008 (4)	0.0008 (4)
C6	0.0122 (5)	0.0154 (5)	0.0169 (5)	0.0007 (4)	0.0009 (4)	0.0003 (4)

### Geometric parameters (Å, º)

**Table:**

N1—C2	1.3401 (15)	C2—C3	1.4069 (16)
N1—C6	1.3510 (15)	C3—C4	1.3793 (16)
N2—C2	1.3919 (15)	C3—H3	0.9300
N2—H2A	0.874 (17)	C4—C5	1.4011 (16)
N2—H2B	0.879 (17)	C4—H4	0.9300
N3—C5	1.4221 (15)	C5—C6	1.3859 (16)
N3—H3A	0.894 (16)	C6—H6	0.9300
N3—H3B	0.898 (17)		
			
C2—N1—C6	118.15 (10)	C4—C3—H3	120.5
C2—N2—H2A	114.3 (11)	C2—C3—H3	120.5
C2—N2—H2B	114.8 (10)	C3—C4—C5	119.83 (11)
H2A—N2—H2B	111.2 (14)	C3—C4—H4	120.1
C5—N3—H3A	111.4 (10)	C5—C4—H4	120.1
C5—N3—H3B	110.4 (10)	C6—C5—C4	117.13 (10)
H3A—N3—H3B	110.2 (14)	C6—C5—N3	122.81 (11)
N1—C2—N2	117.15 (10)	C4—C5—N3	120.01 (10)
N1—C2—C3	121.83 (11)	N1—C6—C5	124.03 (11)
N2—C2—C3	120.90 (11)	N1—C6—H6	118.0
C4—C3—C2	119.02 (11)	C5—C6—H6	118.0
			
C6—N1—C2—N2	−175.08 (11)	C3—C4—C5—C6	0.54 (16)
C6—N1—C2—C3	0.96 (17)	C3—C4—C5—N3	177.98 (11)
N1—C2—C3—C4	−0.66 (17)	C2—N1—C6—C5	−0.51 (17)
N2—C2—C3—C4	175.23 (11)	C4—C5—C6—N1	−0.23 (17)
C2—C3—C4—C5	−0.12 (17)	N3—C5—C6—N1	−177.60 (11)

### Hydrogen-bond geometry (Å, º)

**Table:**

*D*—H···*A*	*D*—H	H···*A*	*D*···*A*	*D*—H···*A*
N2—H2*A*···N1^i^	0.874 (17)	2.183 (17)	3.0541 (15)	175.1 (10)
N2—H2*B*···N3^ii^	0.879 (17)	2.309 (17)	3.1457 (16)	159.3 (10)
N3—H3*A*···N2^iii^	0.894 (16)	2.397 (17)	3.2150 (16)	152.2 (10)
N3—H3*B*···N2^iv^	0.898 (17)	2.593 (17)	3.4803 (16)	170.0 (10)

Symmetry codes: (i) −*x*+1, −*y*+1, −*z*+1; (ii) −*x*+1/2, −*y*+1, *z*+1/2; (iii) *x*, −*y*+3/2, *z*−1/2; (iv) −*x*+1, *y*−1/2, −*z*+1/2.

## References

[bb1] Al-Dajani, M. T. M., Salhin, A., Mohamed, N., Loh, W.-S. & Fun, H.-K. (2009). *Acta Cryst.* E**65**, o2931–o2932. 10.1107/S1600536809044468 PMC2971263 21578508

[bb2] Betz, R., Gerber, T., Hosten, E. & Schalekamp, H. (2011). *Acta Cryst.* E**67**, o2154. 10.1107/S1600536811029412 PMC3213591 22091168

[bb3] Bruker (2001). *SAINT* Bruker AXS Inc., Madison, Wisconsin, USA.

[bb4] Bruker (2005). *APEX2* Bruker AXS Inc., Madison, Wisconsin, USA.

[bb5] Crawford, S., Kirchner, M. T., Blaser, D., Boese, R., David, W. I. F., Dawson, A., Gehrke, A., Ibberson, R. M., Marshall, W. G., Parsons, S. & Yamamuro, O. (2009). *Angew. Chem. Int. Ed.* **48**, 755–757. 10.1002/anie.200803589 19035598

[bb6] Domenicano, A. & Vaciago, A. (1979). *Acta Cryst.* B**35**, 1382–1388.

[bb7] Domenicano, A., Vaciago, A. & Coulson, C. A. (1975). *Acta Cryst.* B**31**, 221–234.

[bb8] Mootz, D. & Wussow, H.-G. (1981). *J. Chem. Phys.* **75**, 1517–1522.

[bb9] Rubin-Preminger, J. M. & Englert, U. (2007). *Acta Cryst.* E**63**, o757–o758.

[bb10] Schwalbe, C. H., Williams, G. J. B. & Koetzle, T. F. (1987). *Acta Cryst.* C**43**, 2191–2195.

[bb11] Sheldrick, G. M. (2003). *SADABS* Bruker AXS Inc., Madison, Wisconsin, USA.

[bb12] Sheldrick, G. M. (2008). *Acta Cryst.* A**64**, 112–122. 10.1107/S0108767307043930 18156677

